# Dietary Cholesterol Supplementation Inhibits the Steroid Biosynthesis but Does Not Affect the Cholesterol Transport in Two Marine Teleosts: A Hepatic Transcriptome Study

**DOI:** 10.1155/2023/2308669

**Published:** 2023-06-05

**Authors:** Ziling Song, Haiyan Xiong, Xiaoxue Meng, Qiang Ma, Yuliang Wei, Yanlu Li, Jian Liu, Mengqing Liang, Houguo Xu

**Affiliations:** ^1^College of Fisheries and Life Sciences, Shanghai Ocean University, 999 Huchenghuan Road, Shanghai 201306, China; ^2^Yellow Sea Fisheries Research Institute, Chinese Academy of Fishery Sciences, 106 Nanjing Road, Qingdao 266071, China

## Abstract

Cholesterol has been used as additive in fish feeds due to the reduced use of fish meal and fish oil. In order to evaluate the effects of dietary cholesterol supplementation (D-CHO-S) on fish physiology, a liver transcriptome analysis was performed following a feeding experiment on turbot and tiger puffer with different levels of dietary cholesterol. The control diet contained 30% fish meal (0% fish oil) without cholesterol supplementation, while the treatment diet was supplemented with 1.0% cholesterol (CHO-1.0). A total of 722 and 581 differentially expressed genes (DEG) between the dietary groups were observed in turbot and tiger puffer, respectively. These DEG were primarily enriched in signaling pathways related to steroid synthesis and lipid metabolism. In general, D-CHO-S downregulated the steroid synthesis in both turbot and tiger puffer. *Msmo1*, *lss*, *dhcr24*, and *nsdhl* might play key roles in the steroid synthesis in these two fish species. Gene expressions related to cholesterol transport (*npc1l1*, *abca1*, *abcg1*, *abcg2*, *abcg5*, *abcg8*, *abcb11a*, and *abcb11b*) in the liver and intestine were also extensively investigated by qRT-PCR. However, the results suggest that D-CHO-S rarely affected the cholesterol transport in both species. The protein-protein interaction (PPI) network constructed on steroid biosynthesis-related DEG showed that in turbot, Msmo1, Lss, Nsdhl, Ebp, Hsd17b7, Fdft1, and Dhcr7 had high intermediary centrality in the dietary regulation of steroid synthesis. In conclusion, in both turbot and tiger puffer, the supplementation of dietary cholesterol inhibits the steroid metabolism but does not affect the cholesterol transport.

## 1. Introduction

Cholesterol, as an essential substance not only for the formation of cell membranes but also for the synthesis of bile acids, steroid hormones, and vitamin D, is the most abundant steroid compound in fish [1, 2]. As vertebrate, fish are able to synthesize cholesterol themselves. Therefore, cholesterol is generally considered as a nonessential nutrient for fish, resulting in only little research interest on the role and mechanism of dietary cholesterol in fish. Limited previous studies in this research area have focused on the effects of dietary cholesterol supplementation (D-CHO-S) on the growth and feed utilization of fish [3–5], and less research has been done regarding the effects on steroid metabolism or other relevant physiological processes. In previous experiments, we have evaluated the effects of D-CHO-S on the growth, tissue biochemical parameters, and expression of lipid metabolism-related genes in turbot and tiger puffer [6], but not the effects on cholesterol homeostasis.

Cholesterol homeostasis is a balance of catabolism, synthesis, intestinal absorption, and biliary secretion. Previous studies have shown that progesterone receptor membrane component 1 (Pgrmc1), cytochrome P450 (CYP), lanosterol 14-alpha demethylase (Cyp51), hydroxysteroid dehydrogenase/ketosteroid reductase, methylsterol monooxygenase 1 (Msmo1), and lanosterol synthase (2,3-oxidosqualene-lanosterol cyclase) (Lss) play key roles in steroid synthesis [7–11]. The cholesterol absorption can be regulated by ATP-binding cassette (ABC) transporters [12]. For example, ABC subfamily A, member 1 (Abca1) serves the efficient cholesterol transport from enterocytes to high-density lipoproteins (HDL) in the serum [13]. Abca1 is also expressed in the liver where it mediates excretion of cholesterol into bile [14]. Other members of ABC family, subfamily G member 5 (Abcg5) and member 8 (Abcg8), are implicated in cholesterol absorption [15]. Member 1 of this subfamily (Abcg1) can also mediate the efflux of free cholesterol to mature HDL [16]. Reverse cholesterol transport (RCT) is known as HDL-mediated transport of cholesterol from peripheral tissues to the liver, where cholesterol can then be removed from via biliary secretion [17–19]. The ABC family also plays important roles in RCT. To more comprehensively investigate the roles of dietary cholesterol in fish physiological processes, in this study, a transcriptomic assay was used to screen the metabolic processes in turbot and tiger puffer, which were responsive to D-CHO-S. In previous studies, it has been observed that D-CHO-S inhibits the synthesis of cholesterol and promotes the synthesis of bile acid [3–5, 20, 21], mainly due to reduced hydroxymethylglutaryl CoA reductase (HMG-CoAr) activity and upregulated cytochrome P450 7A1 (*cyp7a1*) gene expression. However, the available information is still not comprehensive. Nowadays, the transcriptomic analysis technology has become a useful tool in metabolic studies. The transcriptomic analysis may help to elucidate the metabolic progresses and signaling pathways underlying the changes in phenotypic responses.

Turbot (*Scophthalmus maximus*) and tiger puffer (*Takifugu rubripes*) are important aquaculture species [22]. On turbot and tiger puffer, as a follow-up study of Meng et al. [6], the current study is aimed at investigating the physiological responses, in particular the cholesterol metabolism-related ones, of turbot and tiger puffer to D-CHO-S, with transcriptome sequencing. Liver samples from two experimental groups with or without extra cholesterol supplementation were used for the transcriptomic assay. The results obtained from this study will provide basic data for future research in this research area.

## 2. Materials and Methods

### 2.1. Experimental Diets and Feeding Trial

Liver samples were collected from a previous study, and therefore, the detailed procedures for diet preparation and feeding trial have been described previously [6]. Two of the five groups with graded levels of D-CHO-S in the previous study were used in this study. Briefly, the control diet contained 30% fish meal level without fish oil (Table 1) [23–26]. A commercial cholesterol reagent (AR, purity > 95%, Macklin) was supplemented into the control diet at the level of 1.0% to obtain the treatment diet, which was named CHO-1.0. A 10-week feeding experiment with turbot (21 g) and tiger puffer (12 g) was conducted. Each diet was assigned to triplicate polyethylene tanks (200 L, 30 fish per tank). Six fish were randomly collected from each tank for the collection of liver and intestine samples. A whole intestine sample was divided into three parts: anterior intestine (the part near the pyloric caeca), mid intestine (the part near the cecum), and hind intestine (the cecum). All sampling and fish rearing protocols in this study were approved by the Animal Care and Use Committee of Yellow Sea Fisheries Research Institute.

### 2.2. Transcriptome Sequencing and Bioinformatic Analysis

The detailed procedures for RNA isolation, construction of cDNA library, and sequencing have been described in previous publications [27]. Six samples individually collected from the six fish per tank were pooled. Therefore, at last, six pooled samples in total were used for the preparation of six individual cDNA libraries.

Raw reads in fastq format were processed with in-house Perl scripts, during which clean reads can be obtained after low-quality reads, ploy-N-containing reads, and adaptor-containing reads were removed. Meanwhile, other features such as GC content, Q30, and Q20 can be calculated. All the downstream analyses used the clean data.

Prior to the differential expression analysis, the counts of read were adjusted by edge R through one scaling normalized factor. The analysis of differential expression between the two experiment groups was conducted with DESeq2 R (1.20.0). DESeq2 determines the differential gene expression (*P* < 0.05) with a model, which involves the negative binomial distribution.

Gene Ontology (GO) enrichment analysis of the differentially expressed genes (DEG) was conducted with cluster Profiler R, during which the bias of gene length can be corrected. Adjusted *P* < 0.05 indicates significant enrichment by DEG. The Profiler R was also used to test the KEGG enrichment by DEG.

### 2.3. Quantitative Real-Time Polymerase Chain Reaction (qRT-PCR)

Due to the fact that the transcriptomic analysis revealed very few DEG related to cholesterol transport, which was unexpected, qRT-PCR was conducted on selected cholesterol transport-related genes, in order to verify this result, as well as to validate the accuracy of the transcriptomic analysis. The qRT-PCR experiment was conducted for both fish species.

The qRT-PCR methods (see Table 2 for primers), regents (Accurate Biotechnology and Tsingke Biological Technology), and equipment (Roche LightCycler 96, Basel, Switzerland) were the same to our previous publications [6]. The relative mRNA expression was evaluated with the 2^−*ΔΔ*CT^ method [28]. All data were subjected to *T* test for independent samples in SPSS 16.0 for Windows. *P* < 0.05 indicates significant difference. The results are expressed as mean ± standard error.

## 3. Results

### 3.1. Sequence Assembly

In turbot, a total of 133,323,428 and 130,010,624 clean reads were generated for the control group and the CHO-1.0 group, respectively, corresponding to a total clean base of 19.99 and 19.51 G, respectively. For the two groups, the average Q20 and Q30 (the percentage of base with Phred value > 20 and 30, respectively) were 97.68% and 93.76%, respectively. This suggested that the sequencing had high accuracy.

In tiger puffer, a total of 123,437,502 and 127,602,710 clean reads were generated for the control and CHO-1.0, respectively, corresponding to total clean bases of 18.51 and 19.14 G, respectively. For the two groups, the average Q20 and Q30 were 97.83% and 94.03%, respectively. Raw reads were deposited at NCBI's Sequence Read Archive. The accession nos. were PRJNA933270 (turbot) and PRJNA933719 (tiger puffer) (D0 and D1 in the archived files match the control and CHO-1.0, respectively).

### 3.2. Differentially Expressed Genes (DEG) between the Two Experiment Groups

**Figure 1:**
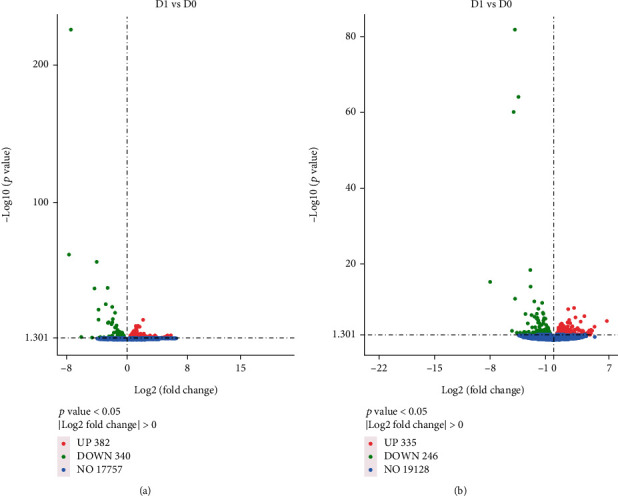
Volcano plot of differentially expressed genes (DEG) in turbot (a) and tiger puffer (b).

In turbot, a total of 722 genes had significantly different expression (*P* < 0.05) between the control and CHO-1.0 (Figure 1(a)). Diet CHO-1.0 upregulated the mRNA expression of 382 genes and downregulated that of 340 genes. In tiger puffer, a total of 581 genes had significantly different expression (*P* < 0.05) between the two treatments (Figure 1(b)). Compared to the control, diet CHO-1.0 upregulated the mRNA expression of 335 genes and downregulated that of 246 genes.

**Figure 2:**
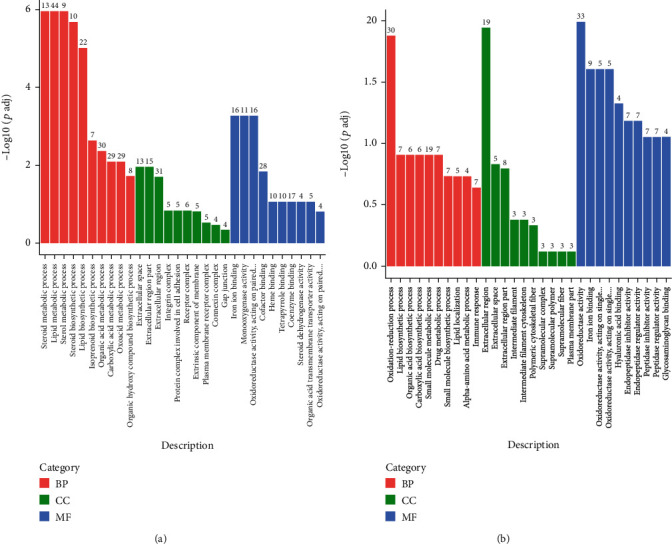
Enriched GO classification of differentially expressed genes (DEG) between dietary groups in turbot (a) and tiger puffer (b). BP: biological process; CC: cellular component; MF: molecular function. Terms not shown in full name in (a): oxidoreductase activity, acting on paired donors, with incorporation or reduction of molecular oxygen; oxidoreductase activity, acting on paired donors, with incorporation or reduction of molecular oxygen, NAD(P)H as one donor, and incorporation of one atom of oxygen. Terms not shown in full name in (b): oxidoreductase activity, acting on single donors with incorporation of molecular oxygen and incorporation of two atoms of oxygen; oxidoreductase activity, acting on single donors with incorporation of molecular oxygen. The number on a certain bar is the DEG number significantly enriched in a certain GO term.

**Figure 3:**
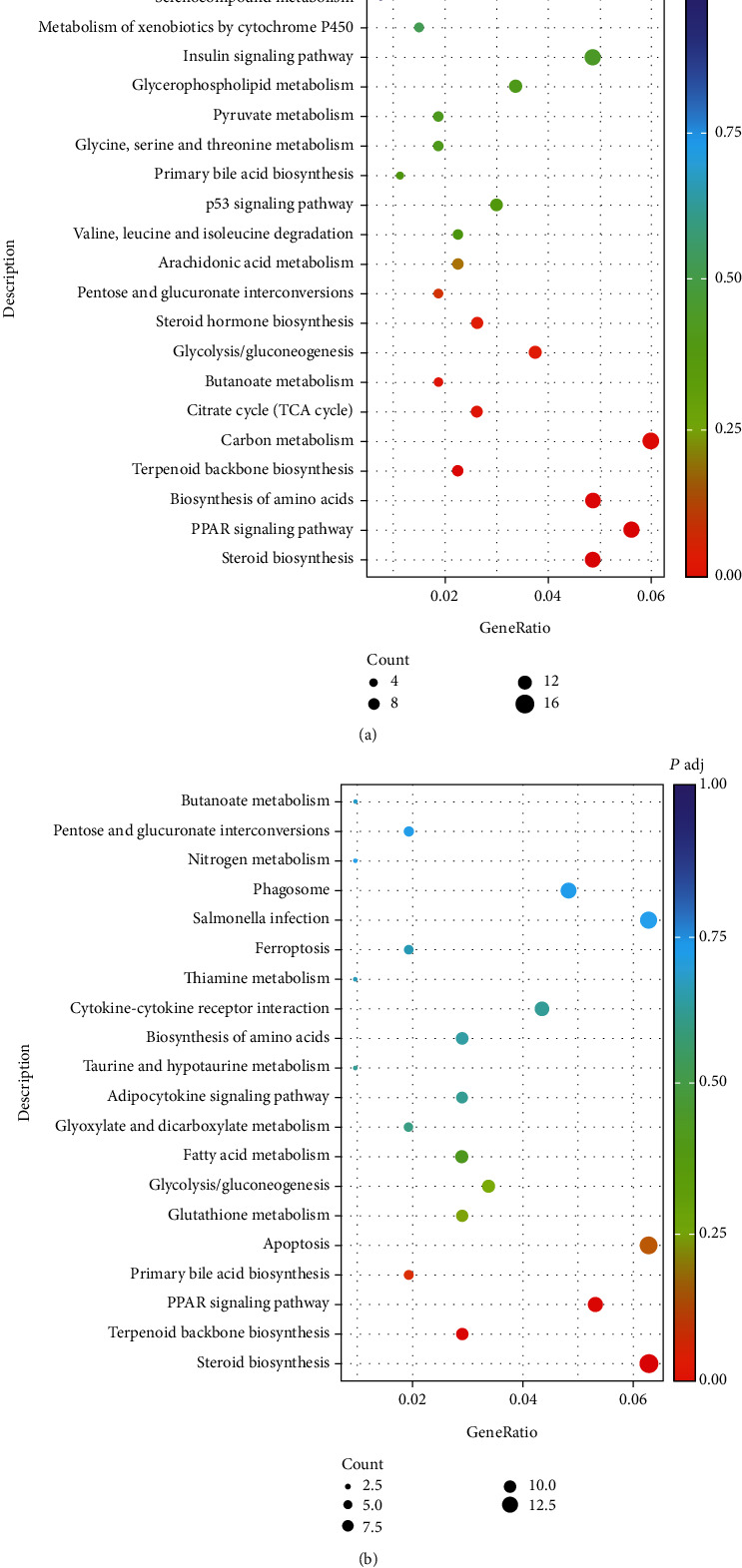
Statistics of KEGG pathway enrichment for differentially expressed genes (DEG) in turbot (a) and tiger puffer (b). The spot size represents the DEG number significantly enriched in a certain KEGG pathway. Padj is the corrected *P* value by multiple hypothesis test.

**Figure 4:**
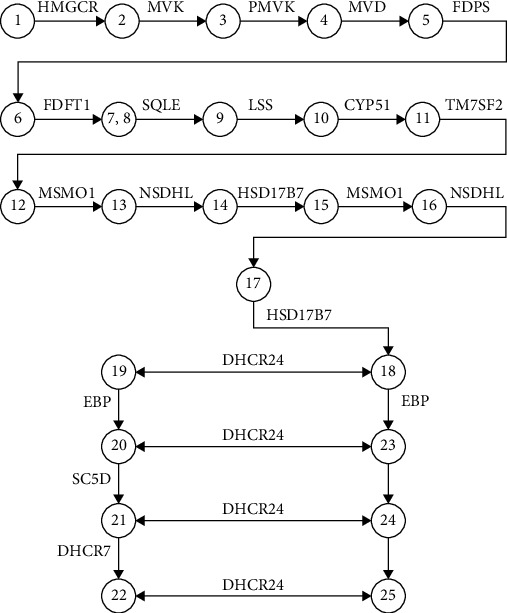
A logical scheme of enzymes and metabolites involved in the cholesterol synthesis pathway [29]. The following metabolites are indicated by numbers: (1) hydroxymethylglutaryl-CoA; (2) (R)-mevalonate; (3) (R)-5-phosphomevalonate; (4) (R)-5-diphosphomevalonate; (5) isopentenyl diphosphate; (6) (2E,6E)-farnesyl-diphosphate; (7) presqualene diphosphate; (8) squalene; (9) (S)-squalene-2,3-epoxide; (10) lanosterol; (11) 4,4-dimethyl-5-alpha-cholesta-8,14,24-trien-3-beta-ol (FF-MAS); (12) 14-demethyllanosterol (T-MAS); (13) 4-alpha-methyl zymosterol-4-carboxylate; (14) 3-keto-4-methylzymosterol; (15) 4-alpha-methyl zymosterol; (16) 4-alpha-carboxy-5-alpha-cholesta-8,24-dien-3-beta-ol; (17) zymosterone; (18) zymosterol; (19) zymostenol; (20) lathosterol; (21) 7-dehydrocholesterol; (22) cholesterol; (23) 5-alpha-cholesta-7,24-dien-3-beta-ol; (24) 7-dehydrodesmosterol; (25) desmosterol.

The GO enrichment suggested that the lipid metabolic process was the primary responsive biological process to dietary cholesterol in turbot (Figure 2(a)), whereas the oxidation-reduction process was the primary responsive process in tiger puffer (Figure 2(b)). The KEGG analysis suggested that PPAR signaling pathway, steroid biosynthesis, biosynthesis of amino acids, and carbon metabolism were the main biological processes responsive to D-CHO-S in turbot (Figure 3(a)), while the PPAR signaling pathway and steroid biosynthesis were the primary responsive processes in tiger puffer (Figure 3(b)). The DEG list also indicates that the steroid biosynthesis and lipid metabolic process may be the primary target processes of D-CHO-S. DEG in steroid biosynthesis with fold changes (FC) greater than four can be seen in Table 3. In turbot, compared to the control, CHO-1.0 downregulated the mRNA expression of squalene epoxidase a (*sqlea*), lanosterol 14-alpha demethylase (*cyp51*), *msmo1*, *lss*, transmembrane 7 superfamily 2 (*tm7sf2*), 24-dehydrocholesterol reductase (*dhcr24*), NAD(P)-dependent steroid dehydrogenase-like (*nsdhl*), sterol-C5-desaturase (*sc5d*), EBP cholestenol delta-isomerase (*ebp*), hydroxysteroid (17*β*), dehydrogenase 7 (*hsd17b7*), 7-dehydrocholesterol reductase (*dhcr7*), and farnesyl-diphosphate farnesyltransferase 1 (*fdft1*). In tiger puffer, CHO-1.0 downregulated the gene expression of *msmo1*, *ebp*, *lss*, *nsdhl*, *dhcr24*, *fdft1*, *dhcr7*, and *sc5d*. Generally, these cholesterol biosynthesis-related DEG can be classified into two categories according to their roles in the enzymatic reaction process (Figure 4) [29]: those far from *dhcr24* and mediating reactions in unidirectional ways, such as *fdft1*, *sqlea*, *lss*, *cyp51*, *tm7sf2*, *msmo1*, *nsdhl*, and *hsd17b7*, and those relevant to *dhcr24*, such as *ebp*, *sc5d*, and *dhcr7*.

### 3.3. qRT-PCR Validation of Gene Expression Related to Cholesterol Transport

**Figure 5:**
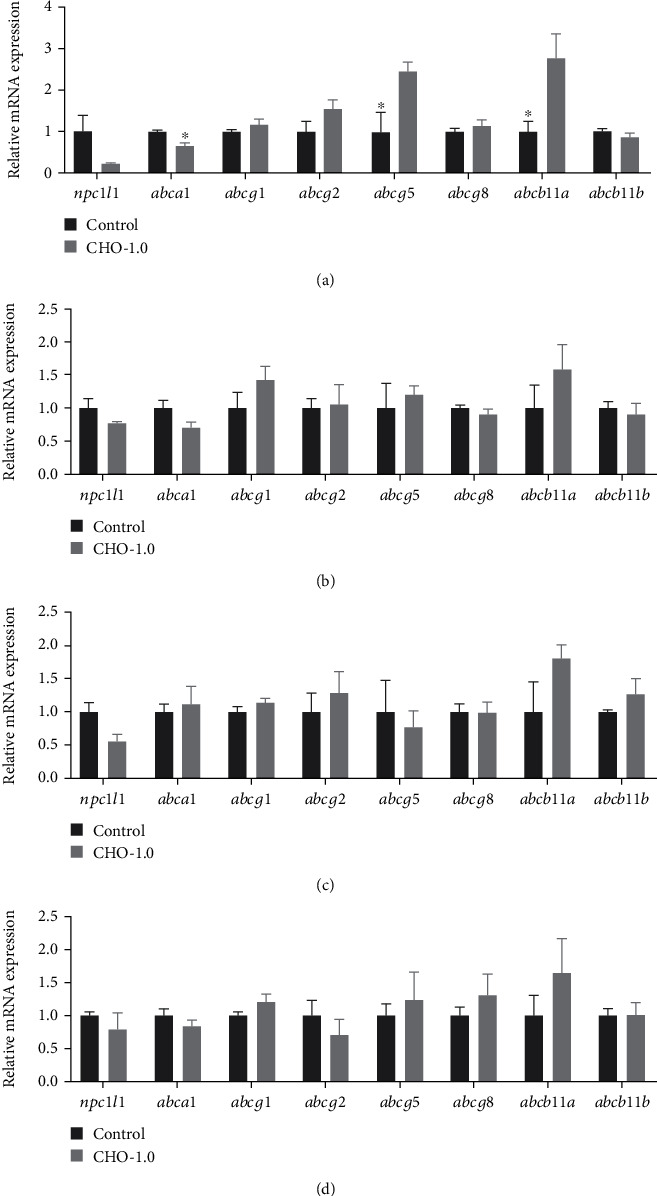
Effects of dietary cholesterol supplementation on the mRNA expression of genes related to cholesterol transport in the experimental turbot (mean ± standard error). ^∗^ represents significant difference between groups (*P* < 0.05): (a) liver, (b) AI: anterior intestine, (c) MI: mid intestine, and (d) HI: hind intestine.

**Figure 6:**
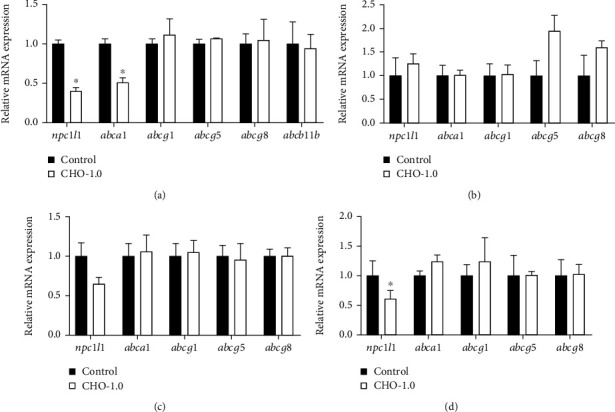
Effects of dietary cholesterol supplementation on the mRNA expression of genes related to cholesterol transport in the experimental tiger puffer (mean ± standard error). ^∗^ represents significant difference between groups (*P* < 0.05): (a) liver, (b) AI: anterior intestine, (c) MI: mid intestine, and (d) HI: hind intestine.

Since few of these DEG are related to cholesterol transport, which is an important component of cholesterol homeostasis, to get more comprehensive information about the regulation of cholesterol transport-related transcription by D-CHO-S, the gene expression of 10 cholesterol transport-related genes, which showed no significant difference between the control and CHO-1.0 from the transcriptomic analysis, was investigated with the qRT-PCR method (Figures 5 and 6).

The results showed that very few changes were observed in response to D-CHO-S in both fish species. Significant changes were observed only in turbot liver and tiger puffer hind intestine. In turbot liver, D-CHO-S significantly downregulated the mRNA expression of *abca1* but significantly upregulated that of *abcg5* and *abcb11a* (Figure 5(a)). In tiger puffer liver, D-CHO-S significantly (*P* < 0.05) downregulated the mRNA expression of *npc1l1* and *abca1* (Figure 6(a)). In tiger puffer hind intestine, D-CHO-S significantly downregulated the transcription of *npc1l1* (Figure 6(d)).

### 3.4. The Protein-Protein Interaction (PPI) Network Construction Based on the Steroid Biosynthesis-Related DEG

**Figure 7:**
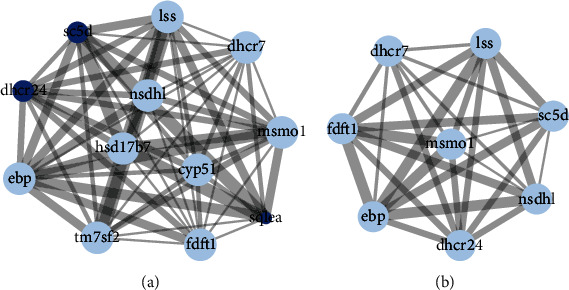
Protein-protein interaction (PPI) network based on the steroid biosynthesis-related differentially expressed genes (DEG) in turbot (a) and tiger puffer (b). Larger node size indicates larger node degree, which is the number of proteins interacting with this node. Brighter node color indicates higher clustering coefficient. Larger edge size indicates higher interacting intensity.

In turbot, *msmo1*, *lss*, *nsdhl*, *ebp*, *hsd17b7*, *fdft1*, and *dhcr7* had higher intermediary centrality than other DEG, indicated by larger node degrees in the PPI network, whereas in tiger puffer, the intermediary centrality of all DEG was the same (Figure 7). In turbot, the clustering coefficient of *dhcr24*, *sc5d*, and *sqlea* was higher, while in tiger puffer, the clustering coefficient of all DEG was the same.

## 4. Discussion

In both species, the steroid biosynthesis was the primary physiological process in response to dietary cholesterol supplementation (D-CHO-S), indicating that this process was sensitive to dietary cholesterol supply. *Msmo1*, *ebp*, *lss*, *dhcr24*, *nsdhl*, *fdft1*, *dhcr7*, and *sc5d* were the main responsive genes shared by the two fish species. Msmo1 is a key protein in the biosynthesis of cholesterol. It mediates the monooxygenation (three-step), which is required for the demethylation of 4 *α*-methylsterols and 4,4-dimethyl [30, 31]. Ebp functions when *Δ*8-sterols are converted to *Δ*7-isomers [32]. The Lss enzyme cyclizes (S)-2,3-oxidosqualene to lanosterol [33, 34]. Dhcr24 reduces the *Δ*-24 double bond of sterol intermediates [35, 36], while Nsdhl decarboxylates the C4 methyl groups of 4-*α*-carboxysterols in postsqualene biosynthesis of cholesterol [37]. Fdft1 is the first enzyme involved in sterol biosynthesis and catalyzes the condensation of 2 farnesyl pyrophosphate moieties to form squalene [33]. Dhcr7 reduces the C7-C8 double bond of cholesta-5,7-dien-3beta-ol and cholesta-5,7,24-trien-3beta-ol, two intermediate metabolites in the cholesterol biosynthesis [38]. Sc5d introduces C5-6 double bond into lathosterol, a dehydrogenation in the biosynthesis of cholesterol [39, 40].

The present result reflected the conservativeness of the steroid biosynthesis process between tiger buffer and turbot. This conservativeness in different fish species has also been observed among mammals, poultry and ruminants [29, 41–43]. The Cancer Genome Atlas in cancer patients showed that the synthesis of cholesterol was mainly mediated by the genes *hmgcr*, *mvk*, *pmvk*, *mvd*, *fdps*, *fdft1*, *sqle*, *lss*, *dhcr24*, *cyp51a1*, *tm7sf2*, *msmo1*, *nsdhl*, *hsd17b7*, *ebp*, *sc5d*, and *dhcr7* [29]. In Jingxing-Huang female chickens, the main responding genes during steroid synthesis were *dhcr24*, *lss*, *msmo1*, *nsdhl*, and *ch25h* [41]. Osteoblasts in mice involved *fdft1*, *sqle*, *lss*, *cyp51*, *msmo1*, *nsdhl*, *sc5d*, *dhcr24*, and *dhcr7* in the process of steroid synthesis [42]. The main genes for cholesterol synthesis in heifers were *hmgcs1*, *hmgcr*, *msmo1*, *mvk*, *mvd*, *idi1*, *fdps*, *lss*, and *dhcr7* [43]. Although these genes involved did not exactly overlap, these findings demonstrate that the steroid synthesis process could be highly conserved among different species.

Among the shared DEG between the two fish species, *msmo1*, *lss*, *dhcr24*, and *nsdhl* might play key roles in the steroid synthesis, because they were more sensitive to D-CHO-S. In turbot, this was confirmed by the PPI network analysis, which showed that *msmo1*, *lss*, and *nsdhl* had high intermediary centrality and *dhcr24* had high clustering coefficient. Nevertheless, in tiger puffer, all DEG had the same intermediary centrality. This could be related to the fact that in tiger puffer, the PPI network was constructed based on less DEG, and thus, there was not enough information to support a high-quality PPI network. Different responsive genes were also observed between turbot and tiger puffer. Four more cholesterol biosynthesis-related DEG, *sqlea*, *cyp51*, *tm7sf2*, and *hsd17b7*, were observed in turbot compared to tiger puffer. Sqlea, which stereospecifically oxidized squalene, is a crucial enzyme in the biosynthesis of steroid [44, 45]. Cyp51 is a key enzyme in the conversion of lanosterol to cholesterol [46]. Tm7sf2 reduces the C14-unsaturated bond of lanosterol [47], and Hsd17b7 is a bifunctional protein in the metabolism of steroid hormone [48]. The difference mentioned above between the two species may be due to the different lipid storage patterns of the two fish species. Cholesterol is synthesized in essentially all tissues of the body, but the liver is presumed to be the primary site. However, for tiger puffer, besides lipid metabolism, the liver also functions as lipid storage organ, which may weaken the function of cholesterol metabolism. Our previous studies have suggested that in tiger puffer, the intestine is probably a lipid metabolism center, but the liver may function as a pure lipid storage organ [49]. Another explanation of this difference could be the fact that tiger puffer body composition has a higher buffer capacity than turbot in response to dietary regulation, which has been indicated by our previous studies [6].

In the present study, the expression of all steroid biosynthesis-related DEG described above was significantly downregulated by D-CHO-S. As shown in Figure 4 [29], all these genes associate with the cholesterol synthesis process and are key enzymes in the different reaction stages of cholesterol synthesis. In both turbot and tiger puffer, downregulation of the steroid biosynthesis genes clearly indicates that the cholesterol synthesis is inhibited when cholesterol was supplemented at 1%. This result was similar to the findings in Atlantic salmon (*Salmo salar*) [50]. The endogenous cholesterol synthesis was probably spared by the exogenous cholesterol supply in the diet. On the other hand, exogenous cholesterol supply may help to guarantee enough substrate for conversion to bile acids. This has been confirmed by our previous research [6], which showed that in both fish species, D-CHO-S downregulated the gene expression of *hmg-coar*, a key enzyme for the biosynthesis of cholesterol [51], but upregulated the *cyp7a1* expression, which has a limiting role in the biosynthesis of bile acid [52].

Cholesterol transport is another important process of cholesterol homeostasis. The intestinal absorption and reverse cholesterol transport (RCT), which is the transport of cholesterol from extrahepatic tissue towards the liver for eventual excretion [17–19], all rely on ATP-binding cassette proteins. Abcg1 catalyzes the efflux of cholesterol from cell to HDL [53], and Abcg2 mediates the transport of intracellular substrate outside the cells [54, 55]. Abcg5 and Abcg8 collaboratively facilitate the transmembrane transport of sterol. They play important roles in the selective excretion of sterol by the liver into bile [56, 57]. Abcb11, which is found in the canalicular membrane, is a primary transporter for the continuous secretion of hepatic bile acids to bile duct [58].

However, the present results showed that D-CHO-S had almost no significant effect on the mRNA expression of these cholesterol transport-related genes. When a certain amount of cholesterol is added to the feed, it is presumed that the cholesterol synthesis decreases and cholesterol excretion capacity increases [51]. This feedback regulation has been observed in another turbot study [20]. Nevertheless, when the cholesterol content is too high, it may be difficult for the fish to fully metabolize them, and the feedback regulation may thereby be impaired. Another explanation of the present result was that the increased cholesterol absorption may offset the increased excretion.

The very few changes in the relative mRNA expression of cholesterol transport-related genes in response to D-CHO-S include upregulation of *abcg5* and *abcb11a* and downregulation of *abca1* and *npc1l1*, mostly in the liver. Downregulation of *npc1l1* expression by dietary cholesterol has also been observed in Atlantic salmon [50]. Because dietary cholesterol is absorbed into small intestinal epithelial cells through Npc1l1 protein, this downregulation could be a typical feedback regulation of fish to reduce the absorption of cholesterol, probably to prevent potential negative influence of excess cholesterol. Abca1 plays a key role in the efflux of intracellular cholesterol to apolipoproteins and the formation of nascent HDL, which facilitates the RCT. The downregulation of *abca1* could indicate an impaired RCT capacity, which was consistent with the reduced HDL-C/LDL-C ratio in the serum [6]. It is well known that LDL helps to carry cholesterol from the liver to the peripheral tissues, while HDL helps to transport cholesterol in an opposite direction [59, 60]. Therefore, the HDL-C/LDL-C ratio can be used as an indicator of RCT [61, 62]. In other turbot studies, when the D-CHO-S level was equal to or greater than 1% (total dietary cholesterol level equal to or greater than 1.25%), the cholesterol easily accumulated in the peripheral tissues [3, 20, 21]. The upregulation of *abcg5* expression by D-CHO-S could be a direct response of Abcg5 to increased cholesterol uptake, considering the roles of Abcg5 in the transmembrane transport of cholesterol across the enterocyte membranes. Anyway, compared to the significant influence on cholesterol biosynthesis, the influence of D-CHO-S on cholesterol transport was minor. Based on the current information, it is difficult to speculate the reasons, and future studies are needed regarding this topic.

## 5. Conclusions

In conclusion, in both turbot and tiger puffer, two important mariculture species, the supplementation of dietary cholesterol inhibits the steroid biosynthesis but seldom affects the cholesterol transport.

## Figures and Tables

**Table 1:** Formulation and proximate composition of the experimental diets (% dry matter).

Ingredients	Control	CHO-1.0
Fish meal^a^	30	30
Corn gluten meal^a^	10	10
Soybean meal^a^	10	10
Casein	10	10
Wheat flour^a^	16.68	16.68
Brewer's yeast	10	10
Mineral premix^b^	0.5	0.5
Vitamin premix^b^	1	1
Monocalcium phosphate	1	1
L-Ascorbyl-2-polyphosphate	0.2	0.2
Choline chloride	0.2	0.2
Betaine	0.3	0.3
Ethoxyquin	0.02	0.02
Mold inhibitor	0.1	0.1
Linseed oil	2	2
Soybean oil	1	1
Rapeseed oil	1	1
Soya lecithin	2	2
*α*-Starch	4	3
Cholesterol^c^	0	1
Proximate composition		
Crude protein	49.75	50.14
Crude lipid	8.34	9.47
Ash	8.02	8.07
Cholesterol	0.11	1.10

^a^The protein content of fish meal, corn gluten meal, soybean meal, and wheat flour was 68.95%, 65.38%, 52.20%, and 15.10%, respectively (dry matter basis), and the lipid content of them was 9.94%, 0.69%, 1.65%, and 1.08% (dry matter basis), respectively. ^b^Vitamin premix and mineral premix, designed for marine fish, were purchased from Qingdao Master Biotech Co., Ltd., Qingdao, China. Generally, the vitamin premix contained retinyl acetate, vitamin D_3_, DL-*α*-tocopherol acetate, menadione nicotinamide bisulfite, thiamine nitrate, riboflavin, vitamin B_6_, cyanocobalamin, D-calcium pantothenate, niacinamide, folic acid, D-biotin, L-ascorbate-2-phosphate, inositol, betaine hydrochloride, yeast hydrolysate, berteroin, and husk powder. The mineral premix contained ferrous sulfate, zinc sulfate, manganese sulfate, cupric sulfate, cobaltous chloride, sodium selenite, calcium iodate, and zeolite powder. ^c^Cholesterol was provided by Shanghai Macklin Biochemical Co., Ltd.

**Table 2:** Sequences of the PCR primers used in this work.

Primer	Sequence (5′-3′)	GenBank reference	PL (bp)	Amplification efficiency (%)
Turbot				
*abca1*-F	AACGACTCTGAAGGTGACCCA	XM_035636643.2	189	93.61
*abca1*-R	AACGACTCTGAAGGTGACCCA			
*abcb11a*-F	ACTTTGGCGTTTGTTGGGTG	XM_035604666.2	170	111.99
*abcba11a*-R	ACTGGCTCCTGGGACACTAT			
*abcb11b*-F	TCGTCTTCCTGACCAACTCGG	XM_035633647.2	170	95.43
*abcb11b-*R	CGAGATCTTGGCCTTGGCGTA			
*abcg1*-F	GCCATGATGGTCTCAGCGAA	XM_035621885.1	101	98.90
*abcg1*-R	GCACAGTCCAGTAAACCCAGA			
*abcg2*-F	CACACTGAGGACCATACCCG	XM_035606728.2	88	104.92
*abcg2*-R	GAGGAAGAAGGCCTCAACCG			
*abcg5*-F	TGGAAGGACGGTACAGAGGA	XM_035606883.2	139	102.63
*abcg5*-R	TGAGGATCTGACGAGTCCGT			
*abcg8*-F	CGTCTGGTCCTGCTTTCAGT	XM_035606882.2	122	97.08
*abcg8*-R	TACGGAACCATGTCACGAGC			
*npc1l1*-F	GCAGTGGCCCTGATCAATCT	XM_035640576.2	97	97.01
*npc1l1*-R	GTGAGGGCTTGGTGCTTAGT			
*β-Actin-*F	GTAGGTGATGAAGCCCAGAGCA	MT023044.1	204	
*β-Actin-*R	CTGGGTCATCTTCTCCCTGT			
*ef1α-*F	TATTAACATCGTGGTCATTGG	KU057926.1	153	
*ef1α*-R	CAGGCGTACTTGAAGGAG			
Tiger puffer				
*abca1*-F	ATCGCTGTTCCTGTCCAAGG	XM_029851394.1	108	108.65
*abca1*-R	CGGGAGATGGTCTGTATGGC			
*abcb11b*-F	ACAACGAACTGCTGGAGAGG	XM_011606221.2	184	121.63
*abcb11b*-R	CGGGAACGCTGACGAATAGA			
*abcg1*-F	GTGCTGAGTCAGGGTCAGTG	XM_029844213.1	84	92.86
*abcg1-*R	GGGGCAGTTGAGTCCAAGTT			
*abcg5*-F	CAGGGTGTTCAGTAGAATCGC	XM_003963888.2	134	100.84
*abcg5*-R	CATAGATGTCGAAAGGGTTGC			
*abcg8-*F	CAAGACAGACTTCCTGGCAAAC	XM_003963887.2	224	99.40
*abcg8-*R	ACAGCGAATGGAGTGAGAGC			
*npc1l1*-F	ATCACACTTTTGGGGCTCGT	XM_011618567.2	157	90.44
*npc1l1*-R	GTCCTGGGTTTGATTCGGGT			
*ef1α-*F	CGACCCTCCATATCTTTCC	XM_011613472.2	84	
*ef1α*-R	CAGCAGCACTTTGCCTTT			
*β-Actin*-F	GAGAGGGAAATCGTGCGTGA	XM_003964421.3	186	
*β-Actin*-R	GAAGGATGGCTGGAAGAGGG			
*rpl13*-F	GTAACAGGTCCACAGAATCCC	XM_003969972	117	
*rpl13*-R	CCTCAGTGCTGT CTCCCTTC			
*rpl19*-F	AAGGGTCGTCAATCTGCGGG	XM_003971891.3	136	
*rpl19-*R	TGGGAGGGATGAACTCTGGG			
*gapdh-*F	ACACCCACTCCTCCATCTT	LOC101067242	100	
*gapdh-*R	TTGCTGTAGCCAAACTCATT			

PL: product length; Abca: ATP-binding cassette subfamily A; Abcb: ATP-binding cassette subfamily B (bile salt export pump, BSEP); Abcg: ATP-binding cassette subfamily G; Npc1l1: Niemann-Pick C1-like 1.

**Table 3:** Differentially (*P* < 0.05) expressed genes (DEG) between the two dietary groups related to steroid biosynthesis. “−” before log_2_FC value represents downregulation in the CHO-1.0 group compared to the control group. FC: fold change.

Gene	Description	Featured ID	log_2_FC	*P* value
Steroid biosynthesis in turbot				
*sqlea*	Squalene epoxidase a	ENSSMAG00000009991	−7.68	8.75*E*-63
*cyp51*	Lanosterol 14-alpha demethylase	ENSSMAG00000004524	−3.98	2.28*E*-57
*msmo1*	Methylsterol monooxygenase 1	ENSSMAG00000002893	−2.57	1.52*E*-38
*lss*	Lanosterol synthase (2,3-oxidosqualene-lanosterol cyclase)	ENSSMAG00000017301	−4.30	3.78*E*-38
*dhcr24*	24-Dehydrocholesterol reductase	ENSSMAG00000016513	−2.79	1.22*E*-26
*tm7sf2*	Transmembrane 7 superfamily member 2	ENSSMAG00000019655	−3.75	1.21*E*-22
*sc5d*	Sterol-C5-desaturase	ENSSMAG00000013863	−1.58	1.89*E*-20
*nsdhl*	NAD(P)-dependent steroid dehydrogenase-like	ENSSMAG00000006484	−2.43	1.57*E*-13
*ebp*	EBP cholestenol delta-isomerase	ENSSMAG00000005050	−1.62	1.10*E*-09
*hsd17b7*	Hydroxysteroid (17-beta) dehydrogenase 7	ENSSMAG00000009286	−1.23	2.34*E*-07
*fdft1*	Farnesyl-diphosphate farnesyltransferase 1	ENSSMAG00000012802	−1.04	0.0002
*dhcr7*	7-Dehydrocholesterol reductase	ENSSMAG00000012834	−0.45	0.0043
Steroid biosynthesis in tiger puffer				
*msmo1*	Methylsterol monooxygenase 1	ENSTRUG00000015761	−4.44	8.37*E*-65
*ebp*	EBP cholestenol delta-isomerase	ENSTRUG00000012886	−2.89	9.61*E*-15
*nsdhl*	NAD(P)-dependent steroid dehydrogenase-like	ENSTRUG00000009297	−2.44	7.90*E*-11
*lss*	Lanosterol synthase (2,3-oxidosqualene-lanosterol cyclase)	ENSTRUG00000013093	−2.11	1.42*E*-07
*dhcr24*	24-dehydrocholesterol reductase	ENSTRUG00000016297	−3.54	1.57*E*-07
*fdft1*	Farnesyl-diphosphate farnesyltransferase 1	ENSTRUG00000012217	−1.21	1.83*E*-06
*dhcr7*	7-Dehydrocholesterol reductase	ENSTRUG00000003928	−2.05	2.69*E*-06
*sc5d*	Sterol-C5-desaturase	ENSTRUG00000012442	−1.29	5.40*E*-05

## Data Availability

Raw data supporting the conclusions of this manuscript will be made available by the authors, without undue reservation, to any qualified researcher.
